# Prevalence of Obesity among Polish Primary Care Population Considered Healthy

**DOI:** 10.3390/nu16172973

**Published:** 2024-09-03

**Authors:** Mateusz Babicki, Karolina Kłoda, Justyna Ledwoch, Wojciech Malchrzak, Sandra Janiak, Filip Krzyżanowski, Tomasz Zieliński, Patrycja Grabska, Dominik Gajowiak, Dagmara Pokorna-Kałwak, Agnieszka Mastalerz-Migas

**Affiliations:** 1 Department of Family Medicine, Wroclaw Medical University, 50-367 Wroclaw, Poland; wojciech.malchrzak@gmail.com (W.M.); filip.piotr.krzyzanowski@gmail.com (F.K.); daga_kalwak@o2.pl (D.P.-K.); agnieszka.mastalerz-migas@umw.edu.pl (A.M.-M.); 2 MEDFIT Karolina Kłoda, 70-240 Szczecin, Poland; wikarla@gazeta.pl; 3 NZOZ Kraków Południe, 30-315 Kraków, Poland; justyna.ledwoch@gmail.com; 4 Department of Family Medicine, Nicolaus Copernicus University in Torun, Collegium Medicum in Bydgoszcz, 85-094 Bydgoszcz, Poland; sandrusssia@gmail.com; 5 NZOZ PROMED A. Szendała, T. Zieliński Lekarze Sp. p., 23-145 Wysokie, Poland; tomasz.zielinski@lzlrp.pl; 6 Przychodnia Lekarska Rodzina Jerzy Rajewski Sp. j., 86-008 Koronowo, Poland; grabskapatrycja@gmail.com; 7 Przychodnia Lekarska Michałek, 62-570 Rychwał, Poland; gajowiak.dominik@gmail.com

**Keywords:** cardiovascular disease, family physician, obesity, prevention

## Abstract

Obesity is a complex disease with numerous health complications, influenced by factors such as genetics, lifestyle, mental health, societal impact, economic status, comorbidities, and treatments. This multicenter study included adults aged ≥35 years referred to a CVD prevention program, where sociodemographic data, anthropometric examinations, laboratory tests, and HLPCQ responses were collected. The study analyzed 1044 patients with a mean age of 47.9 years. Among them, 22.2% (232 patients) were diagnosed with obesity. These patients exhibited higher blood pressure, non-HDL cholesterol, triglycerides, and glucose levels (all *p* < 0.001). A comparative analysis showed that obese patients had significantly lower scores in healthy dietary choices, dietary harm avoidance, daily routine, organized physical exercise, and overall HLPCQ scores. These results indicate that individuals considered healthy were actually living with obesity and its associated complications. Consequently, family physicians should proactively identify patients at risk of obesity using existing programs. The Polish healthcare system urgently needs systemic solutions, including effective health promotion and the creation of obesity prevention programs at an early stage of adult life. These measures are essential to address the growing obesity epidemic and improve public health outcomes.

## 1. Introduction

Obesity is a complex and widespread health problem affecting more and more people in Poland and around the world. Obesity is associated with excessive accumulation of body fat, which leads to metabolic disorders and increases the risk of cardiovascular disease. Although BMI (body mass index) is not an ideal measure, the diagnosis of obesity is still based on a BMI value above or equal to 30 kg/m^2^. A value between 25 and 30 kg/m^2^ is considered overweight, a condition that often precedes the onset of obesity.

According to the World Health Organization, in 2022, one in eight people worldwide were living with obesity [1]. In Europe, the percentage of obese people is about 20% [2]. In Poland, adults living with obesity amount to approx. 9 million, and they have received more than 2 million health services between 2020 and 2022. In our population, obesity is diagnosed more often in men than in women—20% vs. 18% [3].

Obesity increases the risk of many diseases. It is inextricably linked to cardiovascular disease and primarily hypertension, but also ischemic heart disease and atherosclerosis [4].

Obesity often precedes type 2 diabetes, increasing the risk of diabetes many times over [5]. Excess body weight overloads the musculoskeletal system, which promotes joint disease, particularly osteoarthritis. Obesity has also been shown to affect the risk of certain cancers, such as colorectal cancer, pancreatic cancer, and kidney cancer [6]. Obesity-related disease is an independent risk factor that increases the risk of death [7]. It worsens the quality of life of patients, both through mechanical complications and the deterioration of psychological well-being. In contrast, weight reduction significantly improves quality of life [8,9]. Therefore, a lack of diagnosis or an incorrect diagnosis of obesity is associated with failure to implement appropriate treatment methods—both pharmacological and non-pharmacological. This can carry many health consequences, including the development of chronic conditions such as type 2 diabetes, hypertension, etc. [6,7,8].

The main risk factors for the development of obesity are related to genetics and epigenetics, lifestyle, mental health and stress levels, the impact of society, the economic status of the country and the individual, comorbidities, and the treatment used [10,11]. Lifestyle is important in the prevention or development of obesity-related disease, and in its treatment if it occurs. Attention is paid to the pattern and style of eating, i.e., where meals are eaten or self-cooked [12]. This is overlaid with psychological factors, i.e., eating as a way to cope with excessive stress, and the presence of eating disorders [13]. Another area is physical activity. Too little activity contributes significantly to weight gain [14].

Today, practicing a healthy lifestyle is hampered by the media promotion of unhealthy food, underdeveloped infrastructure to encourage physical activity, and in the case of some societies, economic barriers [15]. Knowledge of risk factors, with particular emphasis on those resulting from lifestyle, allows us to identify strategies for dealing with obesity. Both locally and nationally, it is worthwhile to educate the public about appropriate lifestyles so as to effectively prevent obesity, treat obesity appropriately, and maintain remission after weight loss [16]. Government action is also of some importance. Introducing regulations mandating proper food labeling, limiting access to unhealthy foods in schools, or even additional taxation on certain products can support the development of healthy habits [17,18]. Despite the continuous development of knowledge in the field of obesity, it is still necessary to search for factors that influence the differential response to given interventions. It is also unclear which interventions have long-term benefits and which only provide short-term weight loss. It is important to take into account psychosocial factors as well as cultural or socioeconomic differences, and for modern strategies using advanced technology and tools, conclusions and recommendations made in earlier studies should be interpreted with caution.

As obesity is a prevalent, complex and multifactorial disease which results in numerous complications and other diseases, its prevention and early detection are key activities for primary healthcare providers [17,18]. However, due to the lack of systemic organization, the report of the Supreme Audit Office published in March this year showed that Polish family doctors indicate a lack of time and a lack of competence to manage patients with obesity. These are identified systemic and educational gaps that require immediate addressing. To attend to these gaps, multi-level and multi-sectoral involvement is needed. The first step is developing a complex strategy towards obesity prevention and early diagnosis. Such an approach should also involve scientific societies and government entities.

We hypothesize the following: (1) Obesity is a common problem among adults without chronic conditions. (2) Patients with obesity will be characterized by metabolic abnormalities. (3) There is a relationship between lifestyle and obesity. The aim of this study was to analyze the population of PCP patients treated as healthy and referred to a cardiovascular disease (CVD) prevention program in terms of the prevalence of obesity and its metabolic complications among them, as well as their lifestyles, assessed by the HLPCQ.

## 2. Materials and Methods

This is a multicenter study of adults ≥ 35 years referred to the CVD prevention program. The CVD program is a nationwide preventive program dedicated to people aged 35 to 65 who have not yet been diagnosed with cardiovascular diseases, diabetes and chronic kidney disease. Implementation of the program can be carried out by either a PCP or a nurse practitioner.

The inclusion criteria included the following:

- No previously diagnosed cardiovascular conditions and/or diabetes and/or chronic kidney disease;
- Age 35–65 years;
- Consent to participate in the study;
- Laboratory tests performed within the last 4 weeks: lipidogram (total cholesterol, HDL, LDL, non-HDL, triglycerides) and fasting glucose levels.

The patients had to meet all 4 criteria.

The data collection period included patient visits from September 2022 to May 2023. The survey was conducted in 10 PCP units, 6 of which were located in urban areas and 4 in rural areas.

The medical visit consisted of several stages. The first involved the collection of medical history with an assessment of basic sociodemographic data: age, gender, education level, occupation, and place of residence. Also assessed were certain lifestyle parameters such as cigarette smoking (current, past, passive smoking, and no smoking) and physical activity. In addition, the physical activity assessment distinguished between moderate and vigorous physical activity. Moderate physical activity was defined as that which leads to faster breathing and a faster heartbeat (e.g., carrying lighter weights, cycling). Intense physical activity was defined as one that induces very fast breathing and a very fast heartbeat (e.g., carrying heavy objects, kicking the ground, aerobics, fast running, fast cycling). The weekly number of minutes devoted to a specific physical activity was also determined. The family burden of heart attack and stroke in either the mother or the father was also determined. The next stage involved analyzing the laboratory results obtained: a lipidogram (total cholesterol, HDL, LDL, non-HDL, triglycerides) and fasting glucose levels.

The next stage of the medical visit involved taking anthropometric measurements—weight, height, and waist circumference. Waist circumference was measured at the midpoint between the lower edge of the rib arch and the highest point of the hip plate at the median axillary line, according to official recommendations. Based on weight and height, BMI was calculated. On the basis of BMI, patients who met the criterion for the diagnosis of obesity—BMI ≥ 30—were singled out [19].

The final stage of this study involved the patient completing the Healthy Lifestyle and Personal Control Questionnaire (HLPCQ). This is a tool that was initially developed in Greece and was validated and adapted for the Polish population in 2021. The questionnaire consists of 26 single-answer questions based on a 4-point Likert scale: 1—never or rarely; 2—sometimes; 3—often; 4—always. Of the total HLPCQ questions, 12 relate to eating habits, 2 to physical activity, 4 to social support practices and positive thinking, and 8 to daily time management. In addition, the scale has 5 subscales that include the following:

(1) Healthy dietary choices (questions 1, 3, 4, 5, 13, 14, and 16); max points—28.
(2) Dietary harm avoidance (questions 8, 9, 10, and 11); max points—16.
(3) Daily routine (questions 2, 6, 7, 12, 15, 17, 19, and 22); max points—32.
(4) Organized physical exercise (questions 20 and 23); max points—8.
(5) Social and mental balance (questions 18, 21, 24, 25, and 26); max points—20.

The maximum possible number of points to be scored on the scale is 104, and the higher the score, the better the lifestyle. In addition to the total number of points for the entire scale, it is also possible to separately assess the previously mentioned subscales [20].

## 3. Statistical Analysis

The variables analyzed were qualitative and quantitative. The Shapiro–Wilk test was used to assess the normality of distribution. The Chi-square test was used to compare qualitative variables. For quantitative variables, the non-parametric Mann–Whitney U test or Kruskal–Wallis test was used. In addition, Spearman’s correlation was used to establish correlations between age, BMI, laboratory results, and individual subscales of the HLPCQ. Statistical significance was assumed at the level of <0.05. Calculations were performed using Statistica 13 software by TIBCO Software Inc. (Palo Alto, CA, USA).

## 4. Results

### 4.1. Description of the Study Group

The study included 1044 patients with a mean age of 47.9 ± 9.3 years, of whom 64.4% were women and 43.2% were residents of cities with >500,000 residents. In the analyzed group, 248 patients (23.8%) smoked cigarettes, and 60.4% of patients declared some kind of physical activity. After taking into account the criterion of obesity, 232 patients (22.22%) met the criterion for its diagnosis. It was shown that obesity was diagnosed less often in white-collar workers (*p* < 0.001) and those with a higher education (*p* < 0.001). No differences were shown in relation to gender and declared physical activity. A detailed description of the study group and a comparison between patients with and without obesity is presented in Table 1.

### 4.2. Comparison of Blood Pressure Measurements and Metabolic Parameters

Those living with obesity had higher values in blood pressure measurements (*p* < 0.001). Moreover, these subjects also had higher non-HDL cholesterol (*p* < 0.001), triglycerides (*p* < 0.001), and glucose (<0.001) compared to those without diagnosed obesity. There were no differences in total cholesterol (*p* = 0.336) and LDL cholesterol (*p* = 0.098). In addition, an analysis comparing with obesity, overweight, and normal-weight patients showed significantly worse biochemical parameters in the overweight and obese groups compared to the normal-weight group. The results are shown in Table S1, which is Supplementary Materials. A detailed comparison of blood pressure values and laboratory tests is shown in Table 2.

### 4.3. Obesity and Selected Lifestyle Parameters

A comparative analysis of subjects with and without obesity showed that patients with obesity-related disease scored significantly lower on subscales assessing both healthy dietary choices, dietary harm avoidance, daily routine and organized physical exercise, and on the final HLPCQ score. A detailed summary is shown in Table 3.

The correlation analysis showed that for the total population analyzed, regular physical activity correlated with higher HDL cholesterol (r = 0.077, *p* = 0.013) and lower non-HDL (r = −0.093, *p* = 0.003) and triglycerides (r = −0.082, *p* = 0.008). There was also a negative correlation between healthy dietary choices and levels of LDL cholesterol (r = −0.080, *p* = 0.011), non-HDL cholesterol (r = −0.097, *p* = 0.002), and triglycerides (r = −0.071, *p* = 0.023). Moreover, it was shown that, with the exception of social and mental balance, BMI value correlated negatively with the other subscales of the HLPCQ. A detailed summary is shown in Table 4.

Interestingly, among patients with obesity, the correlation analysis showed only a positive correlation between age and healthy dietary choices, dietary harm avoidance, daily routine, and final HLPCQ score.

For patients without obesity, a negative correlation was found between regular physical activity and total cholesterol, LDL, triglycerides, and blood pressure. Convergent correlations included the effect of healthy dietary choices. A summary of the collation results for patients with and without obesity is presented in Tables S2 and S3, which are part of the Supplementary Materials.

## 5. Discussion

In this study, we hypothesized that a population from Poland, assumed to be healthy and thus subjected to a CVD prevention program, is in fact living with obesity and its metabolic complications. We indeed confirmed this disease in 22.22% of the studied individuals. The obtained result is in agreement with other studies from our population [21]. We also explored multiple associations between laboratory parameters and lifestyle choices in relation to obesity, learning that the research questions raised in this study do not have easy and simple answers [22,23].

The analysis based on data from 3663 population-based studies showed that from 1990 to 2022, the age-standardized prevalence of obesity increased in 90 percent of countries worldwide [24]. According to the data collected for NHANES (National Health and Nutrition Examination Survey) between 1998 and 2020, the age-adjusted overall prevalence of obesity in the United States increased from 22.9 to 41.9 percent [25]. Contrary to our findings, most studies showed a higher prevalence of obesity in females [24,26,27]. However, in the data from NHANES 2017–2020, there was no significant difference in the prevalence of obesity between men and women overall or by age group [28]. In the gender analysis in the WHO European Regional Obesity Report 2022 based on STEPwise Survey (STEP) data, inequalities in levels of overweight and obesity between men and women were observed. What is more, they were heterogeneous across socioeconomic determinants such as income, education, employment status, and place of residence [29,30]. The data from our study showed a lower prevalence of obesity among people with higher educational status and white-collar workers. The data on obesity and educational status linkage seem to be conflicting. Some studies show a lower prevalence of obesity among people (especially women) with a higher educational status [31,32,33,34,35], whereas some show no straightforward and significant connections [36,37]. The heterogeneousness of data in this area may be due to more socioeconomic determinants, as aforementioned in the WHO European Regional Obesity Report 2022 [29].

According to WHO data, noncommunicable diseases were responsible for 90% of deaths and 85% of years lived with disability (YLDs) in the WHO European Region in 2021 [38]. Obesity is one of the key risk factors for many noncommunicable diseases and, of course, disease itself. Obesity is linked to an increased risk for cardiovascular diseases, heart failure [39], at least 13 types of cancer [40,41,42,43,44,45,46,47,48,49], type 2 diabetes mellitus [50,51], obstructive sleep apnea, chronic kidney disease [52], and depression [53]. It is thought to lead to over 200 diseases and complications. The idea of metabolic healthy obesity is now a myth. The term “metabolically healthy” patients with obesity refers to individuals who do not have clear adiposity-associated cardiometabolic abnormalities [54]. However, there are sufficient data showing that excess adiposity is linked to increased mortality [50,55,56,57,58,59,60].

The decrease in life expectancy among people living with obesity is similar to that seen with smoking [61]. They also experience healthy life years lost due to diabetes and cardiovascular disease [50]. The years of life lost are highest for people who develop obesity at a younger age and live with obesity for longer [50]. What is more, individuals considered metabolically healthy with obesity had a higher risk of coronary heart disease, cerebrovascular disease, and heart failure than normal-weight metabolically healthy individuals [62,63].

In our study, which was conducted in a so-called healthy population according to the CVD prevention program premise, we established that higher blood pressure, serum non-HDL cholesterol levels, triglycerides, and fasting glucose levels are more prevalent in people living with obesity. Our findings are consistent with other studies [64,65,66]. Type 2 diabetes mellitus is strongly associated with obesity [51,65,66,67]. Thus, weight reduction can decrease the risk of type 2 diabetes [68,69]. Obesity is also associated with unfavorable changes in lipid metabolism, which include elevated serum total and LDL cholesterol, very low-density lipoprotein cholesterol, and triglycerides, as well as a reduction in serum HDL cholesterol [66,70,71]. However, it should be noted that a limitation of the above study is undoubtedly the lack of evaluation of parameters such as insulin and HbA1C, which can be early markers of health problems.

As presented earlier, obesity is a condition with multiple comorbidities and risk factors, but is also a complex disease in its pathophysiology. The grounds of obesity are multifactorial, with a significant role of lifestyle. At least two out of four main behavioral factors linked to mortality from noncommunicable diseases are also factors contributing to the development of obesity (physical inactivity, unhealthy diet) [29]. Therefore, in our study, we compared the results from the HLPCQ between people with and without obesity. The individuals living with obesity present with lower results in the subscales assessing healthy dietary choices, dietary harm avoidance, daily routine, and organized physical exercise.

Studies on food patterns in people living with obesity are scarce, which makes this complex issue (with behavioral and socioeconomic contributing factors) hard to assess [29,72]. The concept of obesogenic environments seems to be more accurate in describing the plethora of factors contributing to the development of unfavorable food behaviors [29]. Regarding physical activity, in a meta-analysis of 10 studies, compared with fit individuals of normal weight, unfit individuals had twice the risk of mortality regardless of BMI [73]. This seems to be consistent with our findings (no difference in declared physical activity between individuals with or without obesity). However, physical activity is associated with reductions in cardiovascular disease incidence [74].

As obesity is a multifactorial disease, it needs to be addressed from many angles. Interventions targeted toward individuals as well as preventive measures on national levels should be encouraged, especially with the burden of the COVID-19 pandemic, which has contributed to a rise in overweight and obesity prevalence [75,76].

## 6. Conclusions

The Polish healthcare system needs urgent systemic solutions, including adequate health promotion and the creation of obesity prevention programs at an early stage of life. Family physicians should actively seek out patients at risk of developing obesity using already existing programs. All actions should also include other cardiometabolic risk factors, which accompany the disease of obesity. The knowledge gaps should be addressed through scientific societies, including the Polish Society of Family Medicine and Polish Society of Obesity Treatment.

## Figures and Tables

**Table 1:** Characteristics of the study group, including the division into patients with and without diagnosed obesity.

Variable		Whole Group (N = 1044)	Without Obesity (N = 812)	With Obesity (N = 232)	*p*
Age M ± SD		47.9 ± 9.3	47.4 ± 9.2	50.0 ± 13.3	**<0.001** ^†^
Sex	Female	672 (64.4)	533 (79.3)	139 (20.7)	0.108 ^‡^
	Male	372 (35.6)	279 (75.0)	93 (25.0)	
Type of profession	White-collar worker	464 (44.4)	388 (83.6)	76 (16.4)	**<0.001** ^‡^
	Manual worker	290 (27.8)	230 (79.3)	60 (20.7)	
	Farmer	85 (8.1)	53 (62.4)	32 (37.7)	
	Pensioner	100 (9.6)	67 (67.0)	33 (33.0)	
	Other	105 (10.1)	74 (70.4)	31 (29.6)	
Education	Basic vocational	231 (22.1)	162 (70.1)	69 (29.9)	**<0.001** ^‡^
	Secondary	286 (27.4)	220 (76.9)	66 (23.1)	
	Higher	462 (44.3)	389 (84.2)	73 (15.8)	
	Other	65 (6.2)	34 (52.3)	21 (47.8)	
Place of residence	Village	326 (31.2)	239 (73.3)	87 (26.7)	**0.003** ^‡^
	City of up to 50,000 inhabitants	261 (25.0)	195 (74.7)	66 (25.3)	
	City of over 50,000 inhabitants	457 (43.8)	378 (82.7)	79 (17.3)	
Heart attack and/or stroke in a father under 55		91 (8.7)	66 (72.5)	25 (27.5)	0.207 ^‡^
Heart attack and/or stroke in a mother under 65		48 (4.6)	37 (77.1)	11 (22.9)	0.905 ^‡^
Smoking	Smokes cigarettes	248 (23.8)	191 (77.0)	57 (23.0)	0.907 ^‡^
	Smoked in the past	177 (16.9)	137 (77.4)	40 (22.6)	
	Passive smoking	22 (2.1)	16 (72.7)	6 (27.3)	
	Does not smoke	597 (57.2)	468 (78.4)	129 (21.6)	
Moderate physical activity	I do not do that kind of activity	413 (39.6)	308 (74.6)	105 (25.4)	0.051 ^‡^
	1–2 times a week	211 (20.2)	175 (82.9)	36 (17.1)	
	3–4 times a week	188 (18.0)	153 (81.4)	35 (18.6)	
	> 4 times a week	232 (22.2)	176 (75.9)	56 (24.1)	
Average time of moderate physical activity [min/week].		189.2 ± 420.5	176.3 ± 387.5	234 ± 518.2	0.664 ^†^
Intense physical activity	I do not do that kind of activity	816 (78.2)	628 (77.0)	188 (23.0)	0.361 ^‡^
	1–2 times a week	96 (9.1)	80 (83.3)	16 (16.7)	
	3–4 times a week	73 (7.0)	60 (82.2)	13 (17.8)	
	> 4 times a week	59 (5.7)	44 (74.6)	15 (25.4)	
Average time of intense physical activity [min/week].		37.3 ± 116.8	37.6 ± 117.3	36.4 ± 115.2	0.287 ^†^

*p*—statistical significance; M—mean; SD—standard deviation; N—number; ^†^ Mann–Whitney U test; ^‡^ Chi-squared test. Statistically significant values are in bold with the significance level set at *p* < 0.05.

**Table 2:** Comparison of blood pressure values and metabolic parameters between patients with and without obesity.

Variable	The Whole Group (N = 1044)	Without Obesity (N = 812)	Obesity (N = 232)	*p*
SBP [mmHg] M ± SD	129.4 ± 17.3	127.0 ± 16.4	137.7 ± 17.5	**<0.001 ^†^**
DBP [mmHg] M ± SD	81.5 ± 10.1	80.2 ± 9.8	86.1 ± 9.9	**<0.001 ^†^**
Heart action [beats per minute] M ± SD	74.5 ± 10.8	74.3 ± 10.4	75.1 ± 11.8	0.814 ^†^
Total cholesterol [mg/dL] M ± SD	204.4 ± 40.2	203.8 ± 39.4	206.4 ± 42.7	0.336 ^†^
LDL [mg/dL] M ± SD	124.3 ± 36.7	123.4 ± 36.2	127.6 ± 38.7	0.098 ^†^
HDL [mg/dL] M ± SD	59.2 ± 15.6	61.1 ± 15.6	52.4 ± 14.0	**<0.001 ^†^**
Non-HDL [mg/dL] M ± SD	145.33 ± 40.5	142.7 ±39.7	154.5 ± 42.0	**<0.001 ^†^**
Triglycerides [mg/dL] M ± SD	115.8 ± 64.4	106.5 ± 58.6	145.3 ± 74.3	**<0.001 ^†^**
Glucose [mg/dL] M ± SD	93.5 ± 16.8	91.9 ± 12.6	99.5 ± 25.9	**<0.001 ^†^**

*p*—statistical significance; M—mean; SD—standard deviation; N—number; mmHg—millimeters of mercury; mg/dL—milligrams per deciliter; SBP—Systolic Blood Pressure; DBP—Diastolic Blood Pressure; LDL—low-density lipoprotein; HDL—high-density lipoprotein; ^†^ Mann–Whitney U test. Statistically significant values are in bold with the significance level set at *p* < 0.05.

**Table 3:** Comparison of HLPCQ scores in relation to the prevalence of obesity.

HLPCQ	Whole Group (N = 1044)	Without Obesity (N = 812)	With Obesity (N = 232)	*p*
Healthy dietary choices	17.0 ± 3.9	17.2 ± 3.9	16.5 ± 3.9	**0.028**
Dietary harm avoidance	10.9 ± 2.8	11.1 ± 20.7	10.5 ± 3.0	**0.005**
Daily routine	20.5 ± 5.6	20.8 ± 5.5	19.4 ± 5.7	**<0.001**
Organized physical exercise	4.2 ± 1.8	4.3 ± 1.8	3.8 ± 1.7	**<0.001**
Social and mental balance	12.8 ± 3.0	12.9 ± 3.0	12.5 ± 3.1	0.073
Final result	65.5 ± 12.9	66.3 ± 12.8	62.7 ± 13.3	**<0.001**

Mann–Whitney U Test. Statistically significant values are in bold with the significance level set at *p* < 0.05.

**Table 4:** Correlation between HLPCQ and age, BMI, blood pressure measurements, and laboratory results.

HLPCQ	Healthy Dietary Choices		Dietary Harm Avoidance		Daily Routine		Organized Physical Exercise		Social and Mental Balance		Final Result	
	r	*p*	r	*p*	r	*p*	r	*p*	r	*p*	r	*p*
Age	−0.003	0.915	0.104	**0.001**	0.088	**0.004**	−0.079	**0.010**	0.002	0.939	0.049	0.115
BMI	−0.092	**0.003**	−0.091	**0.004**	−0.115	**<0.001**	−0.142	**<0.001**	−0.057	0.067	−0.130	**<0.001**
SBP [mmHg] M ± SD	−0.107	**0.001**	−0.024	0.439	−0.006	0.858	−0.078	**0.012**	−0.041	0.190	−0.058	0.060
DBP [mmHg] M ± SD	−0.056	0.073	−0.015	0.637	0.186	0.549	−0.037	0.233	0.010	0.759	−0.015	0.632
Heart action [beats per minute] M ± SD	0.015	0.632	−0.005	0.880	0.050	0.105	0.088	0.778	0.088	**0.004**	0.047	0.131
Total cholesterol [mg/dL] M ± SD	−0.058	0.060	0.015	0.625	−0.028	0.373	−0.067	**0.030**	−0.042	0.176	−0.045	0.144
LDL [mg/dL] M ± SD	−0.080	**0.011**	−0.013	0.674	−0.036	0.251	−0.075	**0.016**	−0.071	**0.022**	−0.069	**0.026**
HDL [mg/dL] M ± SD	0.108	**<0.001**	0.097	**0.003**	0.029	0.350	0.077	**0.013**	0.037	0.234	0.084	**0.007**
Non-HDL [mg/dL] M ± SD	−0.097	**0.002**	−0.024	0.444	−0.033	0.285	−0.093	**0.003**	−0.053	0.085	−0.074	**0.017**
Triglycerides [mg/dL] M ± SD	−0.071	**0.023**	−0.031	0.319	−0.022	0.476	−0.082	**0.008**	0.101	0.745	−0.046	0.134
Glucose [mg/dL] M ± SD	−0.041	0.184	−0.002	0.961	0.157	0.613	−0.058	0.060	−0.004	0.904	−0.149	0.631

M—mean; SD—standard deviation. Spearman’s rank correlation; statistically significant values are in bold with the significance level set at *p* < 0.05. Statistically significant values are in bold with the significance level set at *p* < 0.05.

## Data Availability

The datasets presented in this article are not readily available because the data are part of an ongoing study. Requests to access the datasets should be directed to ma.babicki@gmail.com.

## Supplementary Materials

The following supporting information can be downloaded at: https://www.mdpi.com/article/10.3390/nu16172973/s1, Table S1: Comparison of blood pressure values and metabolic parameters between patients with obesity, overweight and with normal weight; Table S2: Correlation between HLPCQ scale and age BMI, blood pressure measurements and laboratory results among patients with obesity; Table S3: Correlation between HLPCQ scale and age BMI, blood pressure measurements and laboratory results among patients without obesity.
