# Supplementary material for: Prevalence of Obesity among Polish Primary Care Population Considered Healthy
Source: Nutrients. 2024 Sep 3;16(17):2973. doi: 10.3390/nu16172973 (PMC11397732; doi:10.3390/nu16172973)
Supplement: Supplementary file 1 [file nutrients-16-02973-s001.zip › nutrients-3159073-supplementary.pdf]

Table S1. Comparison of blood pressure values and metabolic parameters between patients with obesity, overweight and with normal weight.

Variable	Normal weight (N=441)	Overweight (N=371)	Obesity (N=232)	p
SBP [mmHg] M ± SD	122.7 ± 15.5	132.2 ± 16.2	137.7 ± 17.5	<0.001&
DBP [mmHg] M ± SD	78.1 ± 9.3	82.7 ± 9.8	86.1 ± 9.9	<0.001&
Heart action [beats per minute]M ± SD	74.2 ±10.5	74.5 ± 10.3	75.1 ± 11.8	0.646&
Total cholesterol [mg/dl] M ± SD	200.3 ± 39.5	208.0 ± 38.9	206.4 ± 42.7	0.001&
LDL [mg/dl] M ± SD	118.6 ± 36.6	129.1 ± 34.8	127.6 ± 38.7	<0.001&
HDL [mg/dl] M ± SD	65.3 ± 15.3	56.0 ± 14.3	52.4 ± 14.0	<0.001&
Non-HDL [mg/dl]M ± SD	134.9 ± 39.6	152.0 ± 37.8	154.5 ± 42.0	<0.001&
Triglycerides [mg/dl] M ± SD	91.1 ± 46.8	125.1 ± 65.4	145.3 ± 74.3	<0.001&
Glucose [mg/dl]M ± SD	89.7 ± 9.1	94.4 ± 15.5	99.5 ± 25.9	<0.001&

& Kruskal–Wallis test

Table S2. Correlation between HLPCQ scale and age BMI, blood pressure measurements and laboratory results among patients with obesity.

HLPCQ	Healthy dietary choices		Dietary harm avoidance		Daily routine		Organized physical exercise		Social and mental balance		Final result	
	r	p	r	p	r	p	r	p	r	p	r	p
Age	0.236	<0.001	0.211	<0.001	0.270	<0.001	0.078	0.233	0.074	0.262	0.262	<0.001
BMI	0.045	0.502	0.113	0.087	-0.010	0.879	0.029	0.658	0.113	0.086	0.065	0.327
SBP [mmHg] M ± SD	0.029	0.658	0.098	0.140	0.106	0.110	0.071	0.270	0.023	0.732	0.091	0.171
DBP [mmHg] M ± SD	0.061	0.359	0.126	0.059	0.146	0.027	0.051	0.438	0.039	0.558	0.124	0.060
Heart action [beats per minute	0.017	0.802	-0.069	0.291	0.029	0.663	-0.026	0.698	0.084	0.207	0.018	0.786

]M ± SD												
Total cholesterol [mg/dl] M ± SD	-0.018	0.786	-0.045	0.494	-0.071	0.288	-0.015	0.826	-0.039	0.553	-0.057	0.390
LDL [mg/dl] M ± SD	-0.198	0.765	-0.104	0.116	-0.053	0.425	0.013	0.843	-0.068	0.300	-0.067	0.315
HDL [mg/dl] M ± SD	0.021	0.756	0.039	0.550	-0.065	0.328	-0.026	0.691	0.009	0.885	-0.014	0.836
Non-HDL [mg/dl]M ± SD	-0.018	0.787	-0.070	0.290	-0.034	0.606	-0.004	0.957	-0.041	0.537	-0.046	0.488
Triglycerides [mg/dl] M ± SD	0.026	0.692	0.696	0.293	0.069	0.300	0.009	0.899	0.053	0.420	0.067	0.314
Glucose [mg/dl]M ± SD	0.220	0.740	0.037	0.571	0.117	0.078	0.033	0.616	0.048	0.469	0.081	0.224
M – mean; SD – standard deviation;												

Table S3. Correlation between HLPCQ scale and age BMI, blood pressure measurements and laboratory results among patients without obesity.

HLPCQ	Healthy dietary choices		Dietary harm avoidance		Daily routine		Organized physical exercise		Social and mental balance		Final result	
	r	p	r	p	r	p	r	p	r	p	r	p

Age	-0.629	0.073	0.084	0.017	0.052	0.141	-0.104	0.003	-0.011	0.758	0.039	0.911
BMI	-0.106	0.002	-0.092	0.009	-0.068	0.051	-0.128	<0.001	-0.077	0.029	-0.118	0.001
SBP [mmHg] M ± SD	-0.122	<0.001	-0.034	0.338	-0.004	0.920	-0.084	0.016	-0.044	0.214	-0.068	0.052
DBP [mmHg] M ± SD	-0.071	0.044	-0.030	0.391	0.015	0.661	-0.025	0.470	0.018	0.601	-0.021	0.556
Heart action [beats per minute]M ± SD	0.016	0.642	0.021	0.553	0.061	0.083	0.023	0.522	0.092	0.009	0.060	0.086
Total cholesterol [mg/dl] M ± SD	-0.069	0.049	0.038	0.280	-0.011	0.746	-0.079	0.023	-0.041	0.239	-0.039	0.270
LDL [mg/dl] M ± SD	-0.094	0.007	0.022	0.520	-0.024	0.497	-0.093	0.008	-0.068	0.051	-0.064	0.070
HDL [mg/dl] M ± SD	0.115	0.001	0.082	0.020	0.024	0.505	0.071	0.046	0.029	0.402	0079	0.023
Non-HDL [mg/dl]M ± SD	-0.111	0.001	0.005	0.891	-0.017	0.626	-0.102	0.004	-0.050	0.158	-0.066	0.059
Triglycerides [mg/dl	-0.085	0.016	-0.039	0.266	-0.019	0.580	-0.077	0.029	0.013	0.704	-0.050	0.152

] M ± SD												
Glucose [mg/dl]M ± SD	-0.063	0.73	0.002	0.949	-0.009	0.792	-0.078	0.023	-0.019	0.599	-0.038	0.277
M – mean; SD – standard deviation;												
